# Analysis of Antimicrobial Peptide Expression Under Acute and Chronic Alcohol Exposure: A Cross-Sectional Study and a Systematic Review of the Literature

**DOI:** 10.3390/ijms27042026

**Published:** 2026-02-20

**Authors:** Maura Rojas-Pirela, Cristian Herrera-Flores, Pilar Costa-Alba, Daniel Salete-Granado, María-Lourdes Aguilar, David Puertas-Miranda, Beatriz Cicuéndez, María-Ángeles Pérez-Nieto, Candy Pérez-Albornoz, Cintia Folgueira, Alfonso Mora, Guadalupe Sabio, Miguel Marcos

**Affiliations:** 1 Department of Internal Medicine, University Hospital of Salamanca, 37007 Salamanca, Spain; 2 Institute of Biomedical Research of Salamanca (IBSAL), 37007 Salamanca, Spain; 3 Department of Medicine, University of Salamanca (USAL), 37007 Salamanca, Spain; 4 Department of Cardiology, University Hospital of Salamanca, Centro de Investigación Biomédica en Red de Enfermedades Cardiovasculares (CIBER-CV), 37007 Salamanca, Spain; 5 Primary Care Management of Salamanca, Castilla and León Health Service (SACyL), 37007 Salamanca, Spain; 6 Department of Psychiatry, University Hospital of Salamanca, 37007 Salamanca, Spain; 7 Organ Crosstalk in Metabolic Diseases Group, Molecular Oncology Program, Spanish National Cancer Centre (CNIO), 28029 Madrid, Spain; 8 Faculty of Experimental Science, Universidad Francisco de Vitoria, 28223 Madrid, Spain; 9 Grupo Fisiopatología Endocrina, Área de Endocrinología, Instituto de Investigación Sanitaria de Santiago de Compostela (IDIS), Complexo Hospitalario Universitario de Santiago (SERGAS), 15706 Santiago de Compostela, Spain; 10 Department of Physiology, CIMUS, University of Santiago de Compostela, 15782 Santiago de Compostela, Spain; 11 CIBER Fisiopatología de la Obesidad y Nutrición (CIBERobn), 28029 Madrid, Spain

**Keywords:** antimicrobial peptides, alcohol, immune response, altered expression

## Abstract

Alcohol exposure affects immune regulation and tissue homeostasis. Antimicrobial peptides (AMPs) are essential components of innate immunity, not only defending against pathogens but also modulating processes such as inflammation. However, their tissue-specific regulation in response to alcohol remains poorly characterized, particularly in humans after acute intoxication. We evaluated the expression of AMPs in the peripheral blood of patients with alcohol use disorder (AUD, *n* = 9), individuals with acute alcohol consumption (AAC, *n* = 9), and controls using quantitative polymerase chain reaction (qPCR). Additionally, we analyzed AMP expression in selected tissues of mice exposed to chronic ethanol feeding (National Institute on Alcohol Abuse and Alcoholism model for 5 days) and performed a systematic review of AMP regulation in alcohol-related disorders (2005–2025; n = 36 studies, reflecting a limited and heterogeneous body of available evidence). Human cathelicidin antimicrobial peptide (LL-37), lipopolysaccharide-binding protein (LBP), and bactericidal/permeability-increasing protein (BPI) were significantly upregulated in patients with AUD, whereas LL-37 and LBP were significantly upregulated in AAC. In the livers of ethanol-fed mice, LEP2, LCN2, and LBP levels were markedly increased, whereas LL-37 and LEP1 were downregulated. Duodenal tissue exhibited upregulation of DEFB1. In adipose tissue, DEFA2 was significantly increased in peripheral depots, whereas only LCN2 was upregulated in brain tissue. The systematic review demonstrated complex, heterogeneous, and organ-dependent AMP regulation and also highlighted the paucity of human data on AAC, a gap that our study partially addresses. Our results are consistent with the hypothesis that selected AMPs may serve as candidate markers of organ damage or microbial translocation and as possible therapeutic targets, a hypothesis that requires confirmation in larger, adequately powered studies.

## 1. Introduction

Alcohol consumption represents a major global public health concern. Approximately 2.3 billion individuals worldwide consume alcohol [1] and alcohol use disorder (AUD) is a highly prevalent condition that significantly impairs the quality of life and is often associated with comorbidities and disabling symptoms. Alcohol is recognized as the leading cause of premature death and disability among those aged 20 to 39. In 2019, alcohol consumption was responsible for approximately 2.6 million deaths globally [1,2].

Damage caused by alcohol consumption is associated with immune dysregulation, which is primarily driven by oxidative stress and the generation of reactive metabolites [3,4]. Beyond their well-recognized effects on the hepatic and neurological systems, alcohol and its metabolites also alter immune responses and increase susceptibility to infections, compromising epithelial barrier integrity, altering cytokine production, and modulating the expression of antimicrobial peptides (AMPs) [3,5,6].

AMPs are small peptides, typically composed of 12–200 amino acid residues, that can be expressed constitutively or induced by environmental stimuli. These peptides are key effectors of the innate immune system and constitute the first line of defense against pathogens due to their strong antimicrobial activity against a variety of fungi, bacteria, and viruses [7,8]. Beyond their antimicrobial effects, they exhibit antibiofilm, immunoregulatory, and other biological functions [9]. They may also exert anti-inflammatory effects by blocking receptors involved in lipopolysaccharide (LPS) recognition (e.g., CD14), suppressing the release of immune mediators, and, in some cases, directly binding and neutralizing LPS. Additionally, they modulate signaling pathways (such as NF-κB and MAPK) and regulate the expression of immune-related transcription factors [8]. They also act at the local tissue level by modulating processes such as cell migration, chemotaxis, and phagocytosis [8]. Despite their protective role, increasing evidence also suggests that AMPs play a relevant role in systemic inflammatory responses and tissue damage when dysregulated [10]. Some AMPs can enhance innate immune signaling pathways, including Toll-like receptor–dependent responses, promote the release of cytokines and chemokines, and amplify inflammatory processes that, if sustained, may contribute to tissue injury. These proinflammatory functions can be modulated by complex interactions with the microenvironment, cellular context, and disease setting [11]. Chronic ethanol exposure associated with AUD impairs mucosal defense mechanisms, leading to dysbiosis, bacterial translocation, and systemic inflammation [12]. In parallel, acute alcohol consumption (AAC) can transiently suppress AMP expression in epithelial and immune cells, resulting in compromised barrier function [13] and defense mechanisms against pathogens [12,14]. Notably, deficiency of certain intestinal AMPs increases mucosa-associated bacteria and translocation of these to mesenteric lymph nodes and the liver, favoring the progression of ethanol-induced fatty liver disease to steatohepatitis [14].

Given their potential as biomarkers of mucosal integrity, immune competence [15], and systemic inflammation [10], dysregulated AMP expression profiles have been proposed as indicators of inflammation [16,17] and metabolic liver injury [18]. For example, altered circulating AMP levels have been reported in patients with chronic liver diseases [18] and some infectious processes can play a crucial role in the systemic inflammatory response and tissue damage [10]. Additionally, some AMPs, such as LL-37, have been suggested as a biomarker of the severity of inflammatory conditions, such as vasculitis [17].

Accordingly, characterizing AMP expression patterns in alcohol-related conditions may provide translational insights into host–microbe interactions and immune dysfunction in these diseases. In this context, AMP profiles have been proposed as potential candidate biomarkers for early detection, risk stratification, and therapeutic monitoring in patients with alcohol-related liver disease (ALD) [19,20], and as a possible strategy for treating this disease [21].

Despite the key role of the gut-liver-brain axis in alcohol-related diseases and the growing understanding of AMP function in immunity and inflammation, studies investigating AMP expression and regulation in patients with different patterns of excessive alcohol intake remain limited. Most available evidence is derived from experimental or preclinical models, leaving a significant gap in our translational understanding of AMP-mediated mechanisms in humans. Characterizing AMP profiles in individuals with AUD and AAC is therefore crucial, as it may uncover early molecular alterations associated with immune dysregulation, mucosal barrier impairment, and alcohol-induced damage. Such insights may provide hypothesis-generating evidence to support the future identification of novel biomarkers and therapeutic targets for alcohol-related diseases.

In this study, we tested the hypothesis that both acute and chronic alcohol exposure are associated with a tissue-specific modulation of selected AMPs in humans and in a standardized NIAAA chronic binge murine model. For this purpose, we characterized the expression of key AMPs in peripheral blood (PB) samples of patients with AAC and patients with AUD and evaluated the modulation of these peptides in murine tissues subjected to chronic alcohol exposure using a mouse model of chronic-binge ethanol feeding developed by the National Institute on Alcohol Abuse and Alcoholism (NIAAA). By integrating clinical and experimental data, this investigation sought to elucidate the impact of alcohol on AMP expression dynamics and to explore their potential relevance as biomarkers of immune dysregulation and tissue injury in alcohol-associated conditions. Additionally, we conducted a systematic review following the PRISMA 2020 guidelines and querying the PubMed, Scopus, and Web of Science databases to comprehensively assess current evidence on AMP expression and regulation in alcohol-related disorders. Notably, this review addressed a literature landscape currently dominated by murine models with limited human data, underscoring the translational gap that our integrated clinical-experimental approach aims to address, and providing an evidence-based framework for future translational research. Given the exploratory nature of the study and the small sample sizes, we apply a predefined statistical strategy based on non-parametric group comparisons for targeted AMPs, and we interpret statistically significant results as hypothesis-generating signals rather than definitive evidence of clinical effects.

## 2. Results

### 2.1. Characteristics of the Study Cohort

#### 2.1.1. Patients with AUD

Table 1 summarizes the epidemiological and clinical characteristics of the patient group and healthy controls. No significant between-group differences were observed in age or sex distribution. Patients reported a mean daily alcohol intake of 101.25 g (SD = 5.09) and a chronic consumption history of at least five years. Biochemical analyses revealed significantly increased serum concentrations of aspartate aminotransferase (AST), alanine aminotransferase (ALT), and alkaline phosphatase (ALP) in the patient group. Moreover, the patient cohort demonstrated a significant elevation in total leukocyte and neutrophil counts compared with controls.

#### 2.1.2. Patients with AAC

Table 2 details the epidemiological and clinical characteristics of the AAC group and healthy controls. Sex distribution did not differ significantly between groups. AST levels were significantly elevated in AAC patients compared to those in the control group.

### 2.2. Expression of AMPs and LBP in the PB of Patients with Alcohol Consumption

#### 2.2.1. Expression of AMPs and LBP in the PB of Patients with AUD

**Figure 1:**
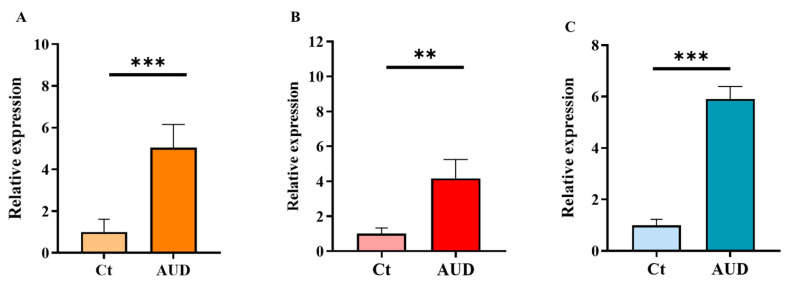
Relative mRNA expression of LL-37, BPI, and LBP in PB from healthy controls (Ct) and patients with alcohol use disorder (AUD). (**A**) Cathelicidin (LL-37), (**B**) bactericidal/permeability-increasing protein (BPI), and (**C**) lipopolysaccharide-binding protein (LBP). Data are presented as relative expression compared to the control group (mean ± SEM). Differences between groups were assessed using the Mann–Whitney U test. *p* < 0.05 was considered statistically significant; *p* < 0.01 (**), *p* < 0.001 (***). Control group *n* = 10, AUD group *n* = 9.

PB samples from patients with AUD exhibited a significant upregulation of AMPs and LBP. Specifically, the relative mRNA expression levels of cathelicidin (LL-37), bactericidal/permeability-increasing protein (BPI), and LBP were markedly elevated in AUD patients compared with control individuals without excessive alcohol consumption (Figure 1).

#### 2.2.2. Expression of AMPs and LBP in the PB of Patients with AAC

**Figure 2:**
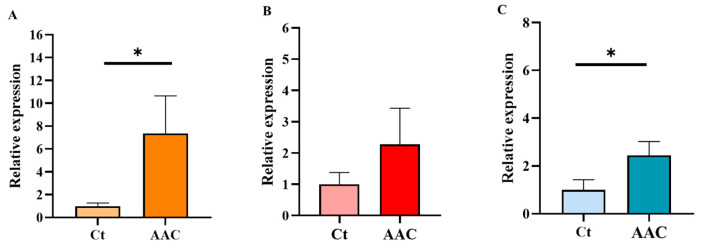
Relative mRNA expression of LL-37, BPI, and LBP in PB from healthy controls (Ct) and patients with acute alcohol consumption (AAC). (**A**) Cathelicidin (LL-37), (**B**) bactericidal/permeability-increasing protein (BPI), and (**C**) lipopolysaccharide-binding protein (LBP). Data are presented as relative expression compared to the control group (mean ± SEM). Differences between groups were assessed using the Mann–Whitney U test. *p* < 0.05 was considered statistically significant; *p* < 0.05 (*). Control group *n* = 9, AAC group *n* = 9.

To assess whether acute alcohol exposure is associated with these AMPs and related innate immune proteins, mRNA expression profiles were also analyzed in an independent cohort of patients with AAC. The results from these analyses showed that only LL-37 and LBP exhibited a significant increase in expression in this cohort (Figure 2). However, although BPI did not reach statistical significance, a trend toward higher expression levels was observed in AAC patients. Taken together, these findings suggest a shared transcriptional response to alcohol intake, regardless of the pattern of ethanol intake.

### 2.3. AMP and LBP Expression in Mouse Tissues Under the NIAAA Model

#### 2.3.1. Expression of AMPs and LBP in Liver Tissue

**Figure 3:**
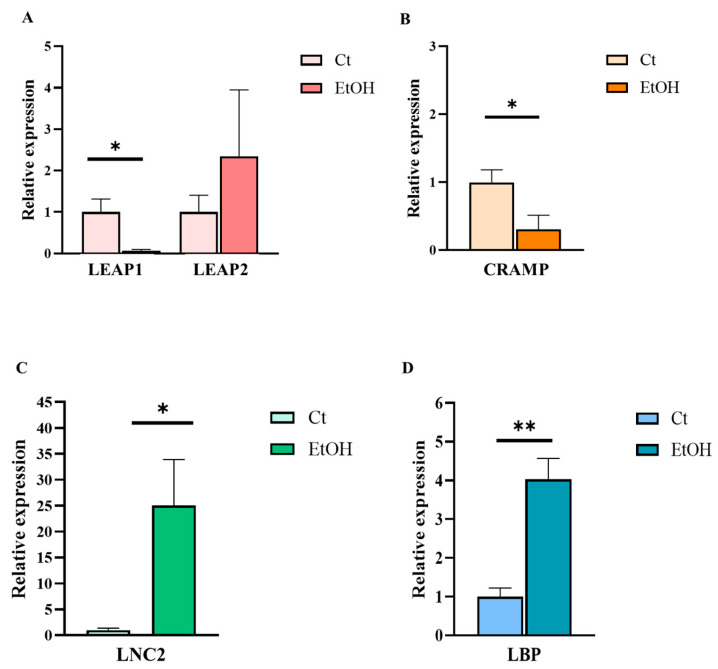
Relative hepatic mRNA expression of AMPs and LBP genes in the NIAAA alcohol-feeding model (EtOH) and control (Ct) mice. (**A**) LEAP1 and LEAP2, (**B**) CRAMP, (**C**) LCN2, and LBP (**D**). Statistical comparisons were performed using the Mann–Whitney U test. Data are presented as mean (standard error of the mean [SEM]). *p* < 0.05 was considered statistically significant; *p* < 0.05 (*), *p* < 0.01 (**). Control group *n* = 5, NIAAA alcohol-fed mice group *n* = 7.

The hepatic expression of several innate immune mediators associated with inflammatory and antimicrobial responses was evaluated, including liver-expressed antimicrobial peptide 1 and 2 (LEAP1 and LEAP2), lipocalin-2 (LCN2), Cathelicidin-related antimicrobial peptide (CRAMP; homologue human LL-37), and LBP. Overall, differences in expression patterns of these mediators were observed in animals fed the ethanol diet compared with the control group (Figure 3). Specifically, a significant increase in LCN2 and LBP expression levels was detected in the livers of alcohol-fed mice. In contrast, CRAMP and LEAP1 expression were reduced relative to control animals, while the increase in LEAP-2 expression was not statistically significant.

#### 2.3.2. Expression of AMPs in Duodenal Tissue

**Figure 4:**
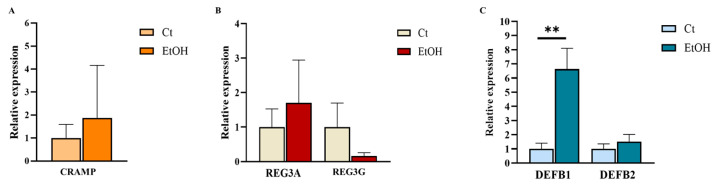
Relative mRNA expression of AMP genes in the duodenum of NIAAA alcohol-feeding model (EtOH) and control (Ct) mice. (**A**) CRAMP: (**B**) REG3A–G and (**C**) DEFB1–2. Statistical comparisons were performed using the Mann–Whitney U test. Data are presented as mean (standard error of the mean [SEM]). *p* < 0.05 was considered statistically significant; *p* < 0.01 (**). Control group *n* = 5, NIAAA alcohol-fed mice group *n* = 7. Large error bars reflect interindividual biological variability.

Consistent with the findings in the liver, a distinct expression pattern of these mediators was detected in the duodenum of mice fed an alcohol-containing diet compared with controls. However, among the AMPs analyzed, only DEFB1 showed a significant increase in expression levels in duodenal tissue from alcohol-fed mice (Figure 4).

#### 2.3.3. Expression of AMPs in Adipose Tissue

**Figure 5:**
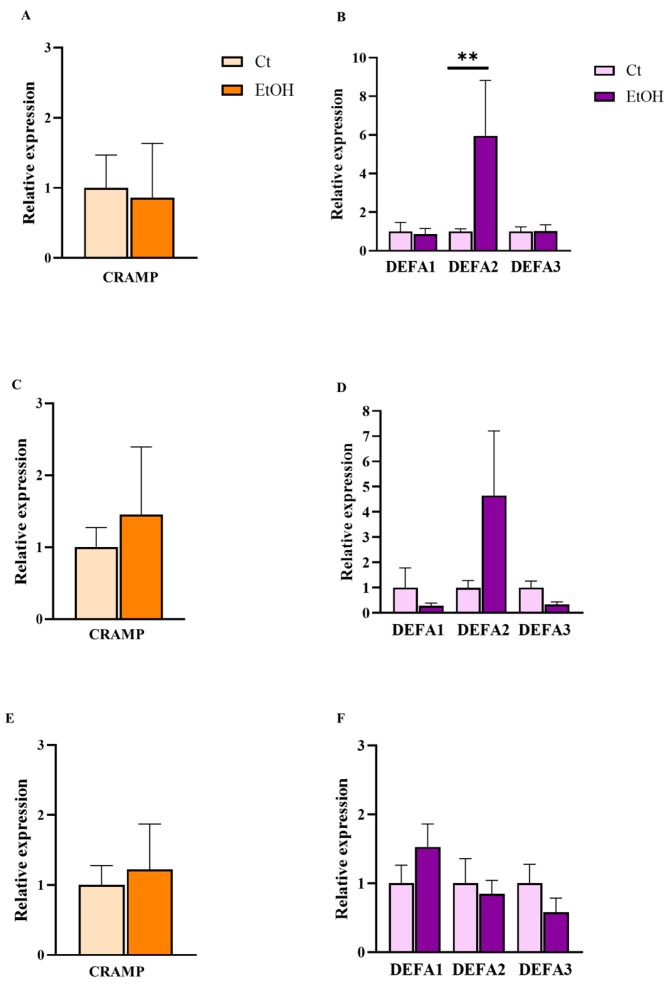
Relative mRNA expression levels of CRAMP and DEFA1–3 in adipose tissue in the NIAAA alcohol-fed model (EtOH) and control (Ct) mice. Panels show CRAMP and DEFA1–3 expression in peripheral (**A**,**B**), visceral (**C**,**D**), and epididymal (**E**,**F**) adipose depots. Statistical comparisons were performed using the Mann–Whitney U test. Data are presented as mean (standard error of the mean [SEM]). *p* < 0.05 was considered statistically significant; *p* < 0.01 (**). Control group *n* = 5, NIAAA alcohol-fed mice group *n* = 7.

In adipose tissue, the expression of AMPs LL-37 and α-defensin family (defensin alpha 1–3 [DEFA1–3]) was evaluated in peripheral, visceral, and epididymal fat depots from control mice and mice fed an alcoholic diet. In peripheral adipose tissue, only DEFA2 showed a significant increase in expression levels in alcohol-fed mice compared with controls (Figure 5). In visceral adipose tissue, a trend toward increased expression of CRAMP and DEFA2 was observed; however, these differences did not reach statistical significance. Conversely, DEFA1 and DEFA3 tended towards decreased expression, with DEFA3 approaching statistical significance (*p* = 0.063). In epididymal adipose tissue, no significant changes were observed in the expression of any of the peptides analyzed.

#### 2.3.4. Expression of AMPs in Brain Tissue

**Figure 6:**
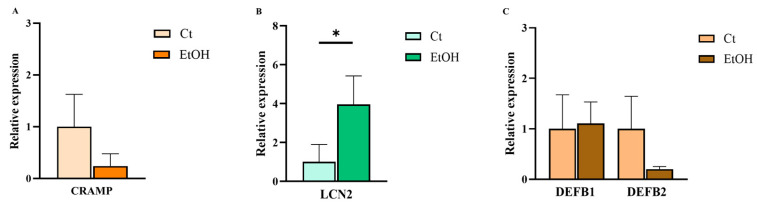
Relative mRNA expression of AMPs in adipose tissue from the NIAAA alcohol-feeding model (EtOH) and control (Ct) mice. (**A**) CRAMP, (**B**) LCN2, and (**C**) DEFB1–2. Statistical comparisons were performed using the Mann–Whitney U test. Data are presented as mean (standard error of the mean [SEM]). *p* < 0.05 was considered statistically significant; *p* < 0.05 (*). Control group *n* = 5, NIAAA alcohol-fed mice group *n* = 7.

Brain tissue analysis revealed that among the antimicrobial peptides LL-37, LCN2, DEFB1, and DEFB2, only LCN2 was significantly upregulated in alcohol diet-fed mice compared with controls. LL-37 and DEFB2 showed a downward trend, while DEFB1 remained unchanged (Figure 6).

### 2.4. Results of Systematic Review

**Figure 7:**
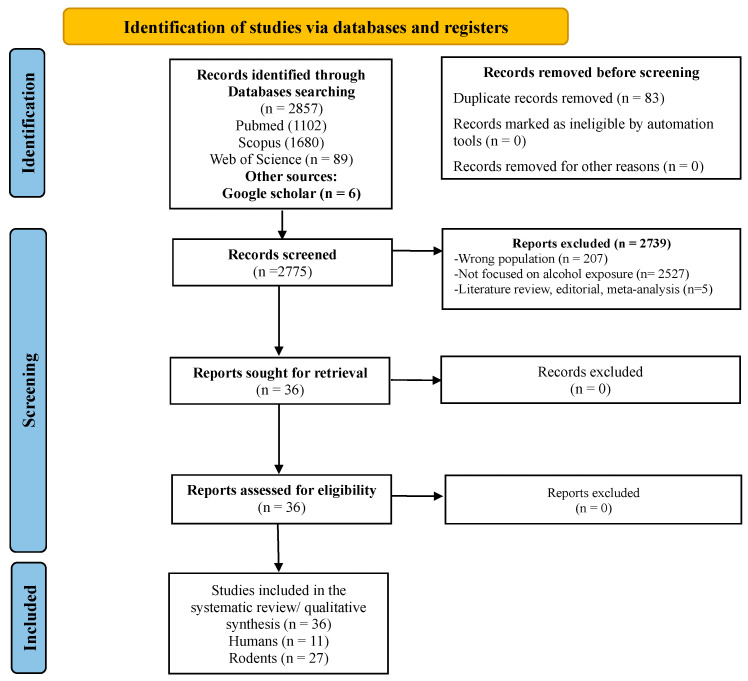
Flowchart of the selection of studies for inclusion in the systematic review.

Our initial search retrieved 2858 articles. After removing 83 duplicates, we excluded 2739 articles based on the title and abstract review, primarily because they were unrelated to the specific interaction between alcohol and AMP expression, or were classified as non-original research (e.g., reviews, conference abstracts). The remaining 36 articles were retrieved in full text, and all were included in the qualitative synthesis (Figure 7), of which 11 investigated human populations and 27 used animal models.

#### 2.4.1. Human Studies Analyzing AMP Expression in Peripheral Blood

Six studies shown in Table 3 [19,20,21,22,23,24] evaluated circulating AMPs in individuals with chronic excessive alcohol consumption, AUD or alcohol-related liver disease and showed heterogeneous results. Most AMPs, such as α-defensins, LEAP-2 or BPI were upregulated in peripheral blood. A single peptide showed reduced circulating levels (e.g., plasma cathelicidin despite increased hepatic expression) [21] and another reported no measurable change (lipocalin-2) [23]. Across studies, several AMPs emerged as disease-specific markers (LEAP-2) [20] or correlated with systemic inflammation, disease severity (α-defensins, REG3A) [19,24] or short-term prognosis (REG3A) [19]. Currently, no studies have investigated the impact of binge drinking or acute ethanol intake on the circulating signature of specific AMPs.

**Table 3:** Human studies included in our systematic review investigating antimicrobial peptides (AMPs) in peripheral blood or urine in excessive alcohol consumption.

Author (Year)	Population	Sample Type	Antimicrobial Peptide	Analytical Technique(s)	Main Findings
Schäfer et al. (2002) [22]	Patient with chronic alcohol misuse (>60 g/day over ≥2 years) with minimal (n =10), intermediate (n = 9) and advanced/cirrhotic (n = 11) liver disease, and healthy controls (n = 11)	Plasma	BPI	ELISA	Markedly elevated; highest in pre-cirrhotic stages
Li et al. (2020) [21]	Patients with AUD (DSM-IV; n = 40) and/or AH (n = 16) and healthy controls (n = 11)	Plasma and liver tissue *	LL-37	ELISA (plasma), qRT-PCR (liver)	Reduced in plasma in active drinkers and abstinent patients with ALD; upregulated in the liver of patients with AH.
Xu et al. (2021) [23]	Patients with alcoholic fatty liver disease (n = 87), NAFLD (steatosis = 83; non-alcoholic steatohepatitis = 277), and healthy controls (n = 40)	Serum and urine	LCN2	Converting phosphor-technology-based lateral flow assay	No significant differences between alcoholic and NAFLD groups
Yang et al. (2021) [19]	Long-term heavy drinkers (>60 g/day for men or > 40 g/day for women, for a minimum of 6 months) with AH (n = 79), heavy drinkers without liver disease (n = 66), and healthy controls (n = 46)	Plasma	REG3A	ELISA	REG3A strongly elevated in AH, decreases with alcohol abstinence and correlates with disease severity, microbial translocation, inflammation, and 30-day mortality.
Liu et al. (2024) [20]	Patients (n = 8) with alcohol-related liver disease (≥ 40 g/day in men or ≥ 20 g/day in women or an ethanol intake > 80 g/day within 2 weeks) vs. patients with MAFLD (n = 26), autoimmune liver disease (n = 8), viral hepatitis (n = 9) and healthy controls (n = 22)	Plasma	LEAP-2	Not reported	Significantly upregulated in ALD vs. MAFLD
Rycyk-Bojarzyńska et al. (2024) [24]	Patients (n = 62) with ALC (European Association for the Study of the Liver criteria) and AUD (AUDIT-C ≥ 3) and 24 healthy controls	Plasma	HNP-1-3	ELISA	Elevated in ALC; associated with NETosis and liver injury and correlated with MELD and mDF scores.

* This study was not included in Table 4 to avoid presenting overlapping results. AH, alcoholic hepatitis; ALC, alcohol-related cirrhosis; ALD, alcohol-related liver disease; AUD, Alcohol Use Disorder; AUDIT, Alcohol Use Disorders Identification Test; BPI, bactericidal/permeability-increasing protein; HNP 1–3, human neutrophil peptides 1–3 (α-defensins); ELISA, enzyme-linked immunosorbent assay; LCN2, lipocalin-2; LEAP-2, liver-expressed antimicrobial peptide-2; MELD, Model for End-Stage Liver Disease; mDF, modified Maddrey’s Discriminant Function; REG3A, regenerating islet-derived protein-3α (also known as HIP [Hepatocarcinoma-Intestine-Pancreas] and PAP [Pancreatitis-Associated Protein]).

**Table 4:** Human studies included in our systematic review investigating antimicrobial peptides (AMPs) in different tissues in alcohol use disorder (AUD).

Author (Year)	Population	Sample Type	Antimicrobial Peptide	Analytical Technique(s)	Main Findings and Implications
Yan et al. (2011) [25]	Patients (n = 10) with chronic alcohol abuse (fulfilling DSM-IV criteria for alcohol dependence and admitted for alcohol withdrawal) and healthy controls (n = 10)	Duodenum	REG3G	qRT-PCR, Western blot	Gene and protein expression were downregulated
Ostaff et al. (2015) [26]	Patients (n = 20) with heavy alcohol use (consuming ≥ 60 g of alcohol per day over a period of at least 6 months) and controls (including those with low-to-moderate alcohol consumption [1–20 g/day]; n = 17)	Gastric antrum, gastric corpus and descending duodenum	HD5, HD6, hBD1, hBD2, hBD4, elafin, lysozyme, sPLA2	qRT-PCR, Immunohistochemistry	Heavy alcohol use increased expression of Paneth cell HD5 and HD6 mRNA in the antrum, but not in the corpus or duodenum. Expression of sPLA2 and lysozyme mRNA remained unchanged. Upregulated HD5 protein levels were independent of intestinal metaplasia. No significant differences were found for β-defensins or elafin
Bajaj et al. (2017) [27]	Cirrhotic patients (n = 20) with active alcohol misuse (AUDIT >8) vs. cirrhotic abstinent for ≥ 6 months (n = 18) and healthy controls (n = 28)	Terminal ileum	HD4, HD5, REG3A, b-defensin, lysozyme, sPLA	qRT-PCR	No differences in AMP expression across groups
Camargo Moreno et al. (2019) [28]	Lung allograft donors (n = 38) with a history of excessive alcohol use (≥ 15 drinks/week for men; ≥ 8 for women, plus phosphatidyl-ethanol blood level >84 ng/mL; n = 18) and non-drinkers (n = 30)	Bronchoalveolar lavage fluid at transplant and 1 month later	LL-37, HNP-1-3, hBD2	ELISA, qRT-PCR	LL-37 increased regardless of infection in lung donors with excessive alcohol use; α-defensins reduced only in infected donors β-defensin-2 protein levels and AMP gene expression remained unchanged Dysregulated levels of LL-37 and α-defensins in the presence of an infection may indicate early graft dysfunction
Hardesty et al. (2022) [29]	Patients with alcoholic hepatitis (n = 40) or alcoholic cirrhosis (n = 40) as defined by histological criteria after exclusion of other liver diseases, and controls (n = 20)	Liver	LL-37	Proteomic and phosphoproteomic analysis	LL-37 expression was upregulated vs. controls, higher in early AH but declined with increasing AH severity

AH, alcoholic hepatitis; hBD, human β-defensin; HD5/HD6, human α-defensins 5/6; ELISA, enzyme-linked immunosorbent assay; HNP 1–3, human neutrophil peptides 1–3 (α-defensins), qRT-PCR, quantitative real-time polymerase chain reaction; REG3A/G, regenerating islet-derived protein 3 alpha/gamma; sPLA2, secreted phospholipase A2.

#### 2.4.2. Human Studies Analyzing Tissue-Specific AMP Expression

Six studies, summarized in Table 3 [21] and Table 4 [25,26,27,28,29], examined AMP expression in gastrointestinal, bronchoalveolar, and hepatic samples from individuals with chronic excessive alcohol consumption and/or alcohol-related liver disease, revealing substantial methodological heterogeneity and discrepant results. Specifically, findings in the digestive tract were inconsistent, with one study reporting upregulation of specific AMPs while others observed downregulation or unchanged levels [25,26,27]. In terms of specific localized responses, gastric a-defensins (HD5, HD6) were induced in the antrum, whereas β-defensins and lysozyme showed no changes [26]. Notably, LL-37 was consistently upregulated in the liver of patients with alcohol misuse and/or related liver disease in 2 studies [21,29].

#### 2.4.3. Rodent Studies Focused on Acute (Binge) Alcohol Exposure and AMP Expression

Six available studies in rodent models of acute alcohol exposure [13,30,31,32,33,34] have primarily analyzed intestinal expression of AMPs (Table 5), with no reports investigating circulating levels or extra-intestinal tissue signatures. Acute alcohol administration triggered AMP alterations in some, but not all, models. Single-dose or short-interval ethanol exposure was sufficient to suppress CRAMP [30]. In contrast, transient increases in REG3β expression were observed in specific acute binge protocols [13,31,33]. Other AMPs (e.g., α-defensins, lysozyme, phospholipase A2 or angiogenins) remained unchanged following isolated ethanol gavage [32,34]. Remarkably, LCN2 displayed a synergistic response, being selectively induced only in the setting of combined alcohol and burn injury [33].

#### 2.4.4. Rodent Studies Focused on Chronic and Binge-on-Chronic Alcohol Exposure and AMP Expression

Similarly, rodent models of chronic alcohol exposure predominantly focused on gastrointestinal tissues, with a notable absence of data regarding circulating AMP levels (Table 6). Across 21 tissue-based studies [21,25,31,35,36,37,38,39,40,41,42,43,44,45,46,47,48,49,50,51,52,53], AMP expression was primarily assessed for Paneth-cell α-defensins (multiple DEFA isoforms) [25,35,36,40,42,44,45,46,51], C-type lectins (REG3B, REG3G) [25,31,35,37,38,39,40,41,43,44,45,47,48,49], and CRAMP [21,49] across the small intestine and colon. Several studies reported downregulation of these peptides at the transcriptional and/or protein level, irrespective of the exposure pattern, which was quite heterogeneous, including continuous intragastric infusion [25,49], the Lieber–DeCarli diet [36,38,39,40,41,43,44,45,46,47,48,50,51], or binge-on-chronic protocols [21,53]. However, some studies suggested that AMP expression may be age-dependent: while aged mice exhibited AMP downregulation, young mice occasionally mounted a compensatory upregulation [37,42]. Moreover, AMP suppression appears to compromise mucosal resilience; for instance, chronic alcohol feeding abolished the protective induction of α-defensins normally observed during colitis [51]. Data from extra-intestinal tissues were limited to a few studies but indicated organ-specific and nonuniform responses, such as CRAMP upregulation in the liver and spleen, contrasted with suppression in lung tissue [21,52]. Collectively, chronic alcohol exposure yielded a dominant phenotype of intestinal AMP suppression across models.

## 3. Discussion

The body of studies examining AMP regulation in response to alcohol is limited and heterogeneous. Our systematic review shows that the regulation of AMPs in response to alcohol remains complex and highly heterogeneous, both in humans and animal models, not only at the biological level but also in terms of methodological aspects such as species, ethanol exposure protocols, sampled tissues, and analytical techniques.

In humans, the heterogeneity of previous studies does not allow us to reach definite conclusions as AMP regulation seems context-dependent and influenced by drinking pattern, disease stage, and the analyzed tissue. Most available studies have assessed individuals with chronic alcohol intake and/or alcohol-related liver disease, using peripheral blood or gastrointestinal biopsies. Of those, only three have specifically enrolled participants with AUD as the primary diagnosis, each with small sample sizes. Across systemic measurements, BPI and α-defensins 1–3 are consistently elevated in the plasma of heavy drinkers or cirrhotic patients, correlating with markers of inflammation, dysbiosis, or liver dysfunction severity. Of note, REG3A is markedly increased in alcoholic hepatitis and decreases with abstinence, supporting its role as a sensitive marker of microbial translocation (REF). In contrast, key lectin AMPs are frequently suppressed in the gut: REG3A and REG3G are significantly downregulated in intestinal biopsies of AUD patients [25,27], potentially compromising mucosal integrity, a pattern that aligns closely with findings in rodent models, and suggests impaired mucosal defense. Some peptides also display discordant regulation across tissues, such as LL-37, which is upregulated in liver tissue but reduced in plasma in alcohol-related liver disease [21]. Although evidence remains limited due to conflicting results and limited data, these findings collectively suggest that AMP expression after chronic ethanol intake reflects a combination of compensatory immune activation and local depletion or dysregulation. Notably, there are virtually no data supporting AMP upregulation in peripheral blood in the context of binge drinking, apart from our own data reported in this manuscript.

Studies focused on rodent models of acute ethanol exposure are also scarce and heterogeneous. Acute alcohol administration often results in modest changes in AMP expression: mouse cathelin-related antimicrobial peptide (CRAMP, a homolog of human LL-37) expression in the ileum is suppressed shortly after binge exposure [30], while other studies report transient upregulation of REG3B following short-term alcohol stress [31]. Overall findings underscore that acute alcohol exposure may exert moderate but context-sensitive dysregulation of key mucosal AMPs, with combined injury amplifying ethanol-related damage [13]. In contrast, several rodent models of chronic or binge-on-chronic alcohol exposure show downregulation of AMPs in gastrointestinal tissues, particularly REG3B, REG3G, and a broad range of α-defensins (e.g., DEFA2, DEFA4, DEFA5) [36,40,44,46]. However, other studies report increased expression of other AMPs such as DEFB1 and DEFB2 [50], no significant change in REG3G [41], and some reports suggest that REG3G regulation differs by age, with downregulation in older mice but preservation or upregulation in younger animals. This age dependence may be particularly relevant because acute binge drinking in humans is most common in adolescents and young adults. Although loss of AMP expression may weaken epithelial antimicrobial defenses [6], facilitating bacterial overgrowth and microbial translocation into the portal circulation, the mixed and heterogeneous pattern of AMP regulation found across studies likely reflects differences in model design, ethanol exposure patterns, sampling sites, and host factors, and it limits direct generalization across studies.

Despite these limitations, animal studies remain highly informative because they highlight AMP modulation as a plausible mediator of gut barrier dysfunction and gut–liver axis injury, and they point to candidate therapeutic targets [21]. Indeed, some models show that restoring AMP expression, particularly intestinal REG3, can reduce bacterial translocation and attenuate liver damage [53]. However, well-designed human studies are needed to validate these signals in defined clinical phenotypes (e.g., AUD without liver disease, alcohol-related hepatitis, cirrhosis) and to determine the role and direction of change of each peptide individually across tissues and disease stages. Overall, our findings reinforce the potential of AMPs as non-invasive biomarkers of disease stage or microbial translocation and as therapeutic targets to mitigate gut–liver axis disruption and inflammatory progression in alcohol-related diseases.

In this context, our new data provide novel evidence that directly addresses the gaps identified in the previous literature by examining AMP responses under both acute and chronic alcohol exposure. Our findings show that excessive alcohol intake, whether a single binge or sustained misuse, modulates the expression of key antimicrobial peptides and related innate immune proteins, reflecting an immunomodulatory effect on host defense mechanisms. Notably, we characterized two AMPs (LL-37 and BPI) and the LPS-binding acute-phase protein LBP in the PB of patients with chronic or acute ethanol intake.

In patients with chronic ethanol consumption associated with AUD, all three mediators were significantly upregulated in PB, suggesting a systemic activation of innate immune responses. A similar but more restricted response was observed in patients with AAC, in whom LL-37 and LBP expression were increased, while BPI showed a nonsignificant upward trend. To our knowledge, these findings provide the first evidence that even acute (binge) alcohol consumption may be associated with AMP upregulation (LL-37) in humans in a similar way to chronic ethanol abuse, a gap previously noted in our review, which is consistent with previous findings showing that acute ethanol binge caused a rapid increase in serum endotoxin, 16S rDNA, and immune-related proteins (LBP and soluble CD14) [54]. Together, this evidence suggests a rapid innate immune response to ethanol exposure and concurrent intestinal translocation. Such a response could enhance immediate protection against bacteria and endotoxins (consistent with the roles of these factors in neutralizing pathogens and endotoxin signaling), but if prolonged, it might also contribute to inflammation. Notably, the systemic AMP upregulation observed in the PB of AUD patients is consistent with prior human studies reporting alcohol-associated modulation of AMPs across tissues [20,24,25,26,27,28]. In contrast, most murine studies have focused on gastrointestinal and hepatic tissue expression, with comparatively limited data on circulating AMPs, and report tissue-specific changes that vary according to ethanol exposure pattern (acute vs. chronic/binge-on-chronic). Together, these observations support a compartmentalized AMP response to alcohol exposure and highlight that PB upregulation may coexist with divergent tissue-level regulation depending on the organ and pattern of alcohol intake.

The biochemical alterations observed in AUD and AAC patients could provide an important clinical context for systemic AMP overexpression. Elevated liver enzymes reflect hepatocellular stress and early tissue injury, while elevated inflammatory hematological parameters (leukocyte and neutrophil counts are indicative of systemic inflammatory activation). Taken together, these alterations are consistent with an inflammatory environment that may promote AMP dysregulation as part of the innate immune response to tissue damage induced by alcohol. In contrast, AAC showed limited biochemical alterations, with an isolated elevation of AST, which parallels the more restricted AMP dysregulation observed in this group, suggesting a possible graded relationship between liver stress and AMP expression.

Parallel to the human findings, our ethanol-fed mouse model revealed a complex, tissue-specific pattern of AMP regulation, suggesting that the effect of alcohol intake on innate immunity varies by organ. In the liver, LCN2 and LBP were significantly upregulated, whereas CRAMP and LEAP1 were markedly downregulated. Unlike the other peptides, LEAP-2 did not show significant changes. The induction of LBP and LCN2 is consistent with their well-established antibacterial functions and their known roles in LPS-driven inflammation and cellular stress responses [55,56,57,58]. LBP, predominantly produced by the liver, facilitates endotoxin recognition and has been implicated in TLR4–NFκB activation in alcoholic liver injury [59,60]. Likewise, LCN2 is a stress-responsive protein that can promote inflammatory signaling and was also elevated in our AUD patients’ circulation, suggesting a coordinated acute-phase response [61,62]. The overexpression of LCN2 in the liver is biologically relevant, as elevated circulating LCN2 can activate its brain receptor (LCN2R), inducing high mobility group box 1 (HMGB1) release and subsequent TLR4–NOX-2–NF-κB signaling, which in turn triggers NLRP3 inflammasome activation and the release of proinflammatory cytokines IL-6 and IL-1β [63].

In contrast, the suppression of hepatic CRAMP and hepcidin may signify the loss of important protective mechanisms. CRAMP is reported to aid in clearing endotoxin and limiting fibrosis in the liver, while hepcidin helps regulate iron to prevent oxidative damage. Their downregulation in chronic alcohol exposure could therefore exacerbate tissue injury by permitting greater endotoxemia and iron-mediated oxidative stress. This divergent regulation, as well as discrepant results with previous studies, highlights that the effect of alcohol on a given AMP can be context dependent. Indeed, we found mRNA expression of LL-37 to be induced in the PB of patients with chronic and acute alcohol exposure yet repressed in the liver of ethanol-fed mice. A prior study by Li *et al.* [21] showed that LL-37 plasma levels were reduced in patients with ALD by ELISA, and upregulated in the liver of both humans and mice by qPCR. Although we can hypothesize that contrasting results are due to methodological differences, particularly the presence of active ethanol intake, this result also reflects how tissue microenvironment and exposure patterns may influence AMP dynamics.

Although differences across studies in the prevalence of active ethanol intake, tissue microenvironment, and exposure patterns may contribute to the contrasting results reported in the literature, our data do not allow us to determine the relative importance or causality of these factors. These should therefore be considered plausible explanations that warrant targeted investigation in future studies.

Although the increase in LEAP-2 lacks statistical significance, LEAP-2 acts through GHSR, a receptor involved in immune modulation, chronic inflammation, and the regulation of energy and metabolic homeostasis via the ghrelin–GHSR1a axis [55,56]. Along with the overexpression of LBP and LCN2, these changes in LEAP-2 may reflect a heightened inflammatory and stress response in the livers of alcohol-fed mice, which, while potentially protective, could also exacerbate tissue injury and promote the progression of alcohol-related liver disease.

Extra-hepatic tissues demonstrated similarly nuanced changes. In the gut, chronic alcohol exposure led to a general dysregulation of DEFB1 in duodenal tissue, with other intestinal peptides showing mixed trends. DEFB1 is crucial for mucosal defense and barrier integrity, so its increase may represent a compensatory response to alcohol-induced barrier disruption, as suggested by other authors [64]. Although CRAMP and REG3 lectins (REG3A and REG3G) did not change significantly, the trends align with their known functions and with previous studies in this field [14]. In murine models, CRAMP upregulation in response to pathogens transiently increases epithelial permeability by promoting endocytic and lysosomal degradation of tight junction proteins, including occludin and claudin-2 [65].

Another novel aspect of our work is our analysis of adipose tissues, an underexplored compartment in AMP research, which revealed differential modulation of AMPs. We found a robust induction of DEFA2 (α-defensin 2) in subcutaneous (peripheral) fat, with a similar trend in visceral fat depots. To our knowledge, this is the first report of alcohol-driven AMP upregulation in adipose tissue. Adipose depots have emerging immune functions [66], and our results suggest that they mount an innate antimicrobial response to ethanol. The coordinated increase of DEFA2 in both peripheral and visceral fat is particularly intriguing, hinting at a depot-specific immune activation that could contribute to local inflammation or host defense. This finding is consistent with limited reports that human adipose can produce defensins to combat infection [67]. On the other hand, we noted no significant changes in adipose CRAMP and a possible mild reduction in DEFA3 in visceral fat, indicating that not all adipose-derived AMPs respond uniformly. Nonetheless, the adipose induction of DEFA2 broadens the potential impact of alcohol on innate immunity, extending it to metabolic tissues and highlighting a previously unrecognized aspect of the alcohol–immune interface.

In the brain, chronic ethanol exposure selectively upregulated LCN2, suggesting a link to neuroinflammation. This is consistent with its established roles in mediating inflammation and oxidative stress in the brain. Elevated central LCN2 expression can contribute to neuronal injury and blood–brain barrier (BBB) dysfunction through multiple mechanisms, including oxidative stress, ferroptosis, and weakening endothelial tight junctions [68,69]. Importantly, LCN2 was upregulated both in the liver and the brain, suggesting a shared inflammatory or stress-related signal along the gut–liver–brain axis [63]. In contrast, the reduction in CRAMP and DEFA2, although not significant, may reflect compromised neuroprotective functions, as these peptides are involved in modulating neuroimmune balance and maintaining neuronal integrity [67,70].

Taken together, our study provides a comprehensive overview of how alcohol exposure modulates AMP profiles across several human and murine tissues. Our findings demonstrate that alcohol exposure induces tissue-specific alterations in AMP expression, impacting both proinflammatory and antimicrobial pathways. The systemic upregulation of LBP and LL-37 in humans, together with selective modulation of hepatic, intestinal, adipose, and brain AMPs in the NIAAA model, underscores the multifaceted effects of ethanol on innate immunity. We also show novel phenomena such as AMP changes in acute drinkers and defensin induction in adipose tissue, which expand the current paradigm of alcohol-related immunity. These novel insights address identified knowledge gaps and highlight AMPs (and related proteins like LBP) as potential biomarkers of alcohol-induced immune dysregulation, which could aid in early detection of ethanol-related damage or risk of complications (e.g., bacterial translocation in the gut or inflammation in the liver and brain). In the long term, strategies to restore or modulate specific AMP pathways might emerge as therapeutic avenues to improve mucosal defenses and hamper the progression of alcohol-related diseases, particularly in the liver and the brain.

Despite the strengths of our integrated analysis, we acknowledge several limitations. The sample sizes of the AUD and AAC patient groups were relatively small, which may limit generalizability and our power to detect subtle changes in AMP expression. In addition, the presence of extreme values within a small cohort may lead to large standard deviations and wide error bars in certain parameters (e.g., GGT), reflecting marked interindividual biological variability and potentially non-normal distributions. Consequently, these findings should be interpreted with caution. Although similar sample sizes have been reported in diverse prior studies investigating transcriptional changes and biomarker discovery in peripheral blood cells of patients with AUD [71,72,73,74], future studies with larger patient cohorts, including diverse populations and longitudinal designs, are warranted to validate these findings and explore additional factors (e.g., sex differences). In addition, group comparisons shown in Table 2 are based on small sample sizes (n = 9 per group) and multiple variables, which limits statistical power and increases the likelihood of chance findings. Therefore, nominal *p*-values are reported for exploratory purposes and should not be interpreted as confirmatory evidence of clinical differences.

An important consideration for interpreting our findings is the substantial heterogeneity which encompassed different species, ethanol exposure patterns, tissues, and analytical methods. In this sense, we have not been able to integrate or combine our results by statistical methods, and our results should be interpreted qualitatively rather than expecting quantitative concordance with individual reports. However, the recurrent observation across heterogeneous studies of increased LL-37/LBP in blood and Lcn2/LBP upregulation in liver/gut strengthens the biological plausibility of the AMP patterns we describe.

While our work partially mitigates the methodological heterogeneity identified in studies included in our systematic review (specifically by using a standardized NIAAA chronic-binge model, well-defined human AAC and AUD phenotypes, and a uniform qPCR-based approach), caution is warranted against over-generalization of our findings. The observed variability in the literature suggests that AMP responses are highly context-dependent; therefore, our results should be interpreted specifically within the exposure patterns and tissues studied here. Future harmonized clinical and experimental studies are essential to validate these AMP signatures across the full spectrum of alcohol-related phenotypes.

Furthermore, given the observational nature of the study, mechanistic studies are needed to elucidate the causal pathways linking alcohol exposure to AMP modulation and the effects on tissue-specific immune responses. Such studies could provide a more comprehensive understanding of how alcohol impacts AMP regulation and innate immune function across tissues.

## 4. Materials and Methods

### 4.1. Subjects

Given their pivotal role in host defense and immune regulation, and the limited knowledge regarding how the duration and pattern of alcohol exposure influence AMP expression, the expression of selected antimicrobial peptides as well as related innate immune proteins (such as lipopolysaccharide-binding protein [LBP]), was evaluated in patients with chronic alcohol exposure associated with AUD and in patients with AAC associated with ethanol intoxication. Biochemical and hematological parameters were included to characterize liver function and the clinical profile of the cohorts, thereby providing context for the interpretation of the immune-related findings.

#### 4.1.1. Sample of Patients with AUD

As previously described [2], this study included nine patients with AUD, diagnosed according to DSM-5 criteria [75], who were recruited from the Alcoholism Unit of the University Hospital of Salamanca (Spain). All patients reported chronic daily heavy ethanol intake (≥100 g/day for at least 5 years). All participants had normal prothrombin time, serum albumin levels, and hemoglobin, and tested negative for hepatitis B and C. Exclusion criteria included other chronic or acute illnesses, polydrug use, and advanced liver disease. Advanced liver disease was ruled out by clinical examination, laboratory testing, and ultrasonography. Participants were excluded if they presented physical signs of chronic liver disease, ultrasonographic abnormalities other than steatosis, or liver transaminase levels 2–3 times above the reference range. Together, these criteria ensured that the AUD participants constituted a clinically homogeneous group, characterized by a uniform DSM-5–based diagnosis, a similar pattern of alcohol consumption, comparable clinical status, and the absence of relevant comorbidities.

Ten healthy volunteers, matched for age and sex, consuming <15 g ethanol/day and exhibiting normal liver function and routine laboratory tests, served as controls. Blood samples for hematological and biochemical analyses, together with PAXgene Blood RNA tubes (Becton Dickinson, Franklin Lakes, NJ, USA), were collected from all participants between 8:00 and 10:00 a.m. following an overnight fast. Written informed consent was obtained from all participants, and the study was approved by the Ethics Committee of the University Hospital of Salamanca (PI2023/07/1389) on 27 November 2023.

#### 4.1.2. Samples from Patients with AAC

Nine adolescents and young adults presenting with moderate to severe acute alcohol intoxication at the Emergency Department of the University Hospital of Salamanca (Spain) were included in this study, as reported previously [2]. Acute alcohol intoxication was defined clinically by signs including ataxia, slurred speech, impaired reasoning, or disorientation, alongside blood alcohol levels >1 g/L and recent consumption of at least five standard drinks (50 g) for men or four (40 g) for women within the six hours preceding admission. In addition, nine healthy controls, age and sex-matched, were included. These individuals reported consuming alcohol only lightly and sporadically, refrained from drinking for 72 h before blood collection, and reported no binge drinking within the last three months. All control participants displayed normal hematological and biochemical parameters and results and reported no chronic or acute medical conditions. Urine toxicology testing was conducted, and those with a history or clinical signs of illicit drug use (except cannabis), illness, or medication use were excluded from the study. Blood samples, including PAXgene Blood RNA tubes, were collected from patients with AAC at hospital admission for ethanol intoxication under an ethics committee–approved deferred-consent protocol. After patients were no longer under the influence of alcohol and able to provide informed consent, written consent for research participation was obtained for the use of samples and data collected at admission. If consent was declined, patients were not included in the study and research samples were destroyed; samples obtained for clinical care were retained and used for clinical purposes. Samples from healthy controls were obtained following an overnight fast and after written informed consent.

### 4.2. Mice, Experimental Conditions, NIAAA Model, and Extraction of Organs

Since each organ plays a distinct role in host defense and inflammatory balance, we assessed AMP expression across multiple tissues to explore potential tissue-specific effects of alcohol exposure. The study focused on AMPs that are selectively expressed in, or play particularly relevant roles within, each tissue, thereby providing a more comprehensive understanding of how alcohol modulates their expression at the local level.

For this study, seven C57BL/6J male mice, aged 8–10 weeks, were maintained under standard laboratory conditions (12 h light/12 h dark, temperature-controlled at 22 ± 2 °C, with ad libitum access to food and water). Five male mice received an isocaloric control diet without ethanol. The NIAAA model was employed to induce ethanol-related liver damage, simulating chronic alcohol consumption, as described previously [76]. The protocol consisted of 5 days of a liquid diet with 5% ethanol, followed by a single oral dose of ethanol (5 g/kg) to induce a binge-like episode. Nine hours after ethanol binge administration, the animals were euthanized in accordance with approved procedures. Tissue samples, including brain, liver, duodenum, and adipose tissue (peripheral, visceral, and epididymal), were collected immediately after euthanasia. The tissues were rapidly washed in PBS, cut into small fragments, and immediately frozen in liquid nitrogen, then stored at −80 °C until further processing. All procedures on animals adhered to EU Directive 2010/63/EU and Recommendation 2007/526/EC regarding the protection of animals used for experimental and other scientific purposes, as implemented under Spanish Law 1201/2005.

### 4.3. RNA Isolation, Reverse Transcription, and Quantitative Real-Time PCR (qRT-PCR)

The expression of mRNA in peripheral blood (PB) was assessed using PAXgene™ Blood RNA Tubes. Total RNA was isolated with the PAXgene™ Blood RNA Kit (QIAGEN, Hilden, Germany) following the manufacturer’s instructions and stored at −80 °C until use. Total RNA from tissues was extracted with the NucleoSpin^®^ RNA kit (MACHEREY-NAGEL GmbH & Co. KG, Düren, Germany) following the manufacturer’s instructions. RNA concentration and purity were evaluated using a NanoDrop ^TM^ 2000 spectrophotometer (Thermo Fisher Scientific, Waltham, MA, USA).

Complementary DNA (cDNA) synthesis was performed from total RNA using the High-Capacity cDNA Reverse Transcription Kit (Applied Biosystems, Waltham, MA, USA). Quantitative real-time PCR (qRT-PCR) was performed on a StepOnePlus™ Real-Time PCR System (Applied Biosystems, Waltham, MA, USA). All qRT-PCR reactions were performed in technical triplicate, and the primer specificity (see Supplementary Table S1) was verified by melt curve analysis. Gene expression was normalized to endogenous controls as follows: serine and arginine-rich splicing factor 4 (SRSF4) for duodenum, brain, and liver samples; hypoxanthine phosphoribosyltransferase (Hprt) for visceral and epididymal adipose tissue; TATA-binding protein (TBP) for peripheral adipose tissue; and 18S ribosomal RNA (18S) for PB. Quantification was performed using the 2^−ΔΔCt^ method, and results are expressed as fold changes relative to control values.

### 4.4. Statistical Analysis

Quantitative variables are presented as mean ± standard deviation (SD) (Table 1 and Table 2), and qualitative variables are reported as absolute (n) and relative (%) frequencies. qRT-PCR data are expressed as mean ± standard error of the mean (SEM), with Ct values normalized to a reference gene. Comparisons between control and patient groups, or between control mice and mice under the NIAAA model, were performed using the Mann–Whitney U test. Statistical significance was defined as *p* < 0.05. All analyses were conducted using GraphPad Prism software (version 10, GraphPad Software, Boston, MA, USA), with additional details provided in the figure legends.

### 4.5. Bibliographic Search and Systematic Review

#### 4.5.1. Literature Search Strategy

A systematic review of the literature was conducted to explore the expression of AMPs in patients with acute or chronic excessive ethanol consumption or in animal models of ethanol exposure. This search aimed to identify current evidence on how alcohol consumption affects AMP expression, regulation, and function across different tissues and biological systems. Two investigators (CH and MRP) independently performed the bibliographic search, with any divergence of opinion resolved by consensus with a third author (MM). This review followed the PRISMA 2020 guidelines for systematic reviews [77]. Three search strategies (three Boolean strings) were developed for three different databases: Scopus, Web of Science (WOS), and PubMed (see Supplementary Materials Section S1). No restrictions were placed on language, sample size, or publication date. We also manually revised the reference lists of obtained publications to identify additional pertinent articles. In addition, the search was complemented by using the Medline “related articles” option and examining review articles on the topic. Additionally, we conducted a manual search using Google Scholar to identify relevant publications that were not indexed in the databases. The literature search was updated to 22 November 2025.

#### 4.5.2. Article Selection and Data Extraction

We included original research articles that examined the expression of AMPs in the context of alcohol-related organ damage using human and/or animal samples, such as blood, plasma, serum, or tissues (e.g., liver). We excluded reviews, letters, conference abstracts, book chapters, and theses. For each eligible study, we collected the following data: authorship, year of publication, human or animal model of ethanol exposure, sample type, AMPs investigated, analytical methodology, and main results. Data were extracted independently by two authors (CH and MRP) and differences were resolved by consensus. The literature was summarized in tables where appropriate.

## 5. Conclusions

In this exploratory study and systematic review, we observed that alcohol exposure, whether acute or chronic, induces complex, tissue-specific alterations in AMP expression in human and animal models. Specifically, chronic alcohol consumption correlated with higher levels of systemic AMPs such as LL-37 and LBP in PB, while selectively modulating hepatic, intestinal, adipose, and brain AMPs. Notably, LCN2 was consistently upregulated in both liver and brain, suggesting a potential link between peripheral and central inflammatory responses. DEFB1 upregulation in the duodenum and DEFA2 induction in adipose tissue highlight compensatory, tissue-specific immune responses. Collectively, these findings suggest that alcohol affects both proinflammatory and protective AMP pathways, positioning AMPs as potential biomarkers and mechanistic mediators of alcohol-induced immune dysregulation. This study provides a comprehensive multi-tissue overview of AMP modulation in response to alcohol, offering new insights into how ethanol alters host defense and tissue homeostasis. The systematic review based on the available evidence suggests that AMP regulation in response to alcohol exposure is complex and heterogeneous. This expression can be influenced by multiple factors, including patterns of alcohol consumption, disease stage, tissue, and host characteristics. Additionally, studies in both humans and animals reveal heterogeneous AMP expression profiles, reflecting a balance between compensatory immune activation and localized dysregulation of mucosal defenses. While chronic alcohol exposure appears to be more consistently associated with AMP alterations in tissues, reports on acute alcohol consumption remain limited, particularly in humans.

Given the limitations of sample size and the cross-sectional design, these results should be interpreted as preliminary evidence. Rather than definitive mechanistic mediators, our findings highlight AMPs as potential candidates for future investigation into alcohol-induced immune dysregulation. Further longitudinal studies with larger cohorts are necessary to validate their utility as candidate biomarkers of organ damage or microbial translocation and potential therapeutic targets.

## Figures and Tables

**Table 1:** Demographic and clinical characteristics of patients with alcohol use disorder (AUD) and controls. Quantitative variables are expressed as mean (standard deviation), and qualitative variables as * number of cases (percentage). AST: aspartate aminotransferase; ALT: alanine aminotransferase; GGT: gamma-glutamyl transferase; ALP: alkaline phosphatase. A *p* < 0.05 was considered statistically significant.

Variable	AUD Patients (*n* = 9)	Controls (*n* = 10)	*p*-Value
Age (years)	53.55 (11.20)	46.90 (11.32)	0.18
Male (n *)/Female (n *)	7 (77.77)/2 (22.22)	6 (60)/4 (40)	0.43
Total bilirubin concentration (mg/dL)	0.85 (0.48)	0.73 (0.28)	0.91
AST activity (U/L)	117.28 (98.65)	18.10 (4.74)	0.049
ALT activity (U/L)	54.77 (39.95)	23.20 (18.05)	0.062
GGT activity (U/L)	348.28 (518.99)	17.70 (11.00)	0.16
ALP activity (U/L)	94.44 (29.80)	60.71 (15.93)	0.017
Proteins (g/dL)	7.36 (0.79)	7.49 (0.42)	0.69
Albumin (g/dL)	4.58 (0.35)	4.75 (0.21)	0.28
Ferritin (ng/mL)	180.11 (109.09)	115.36 (109.47)	0.41
Hemoglobin (g/dL)	16.20 (1.42)	14.91 (1.24)	0.070
Leukocytes (×10^3^ cells/μL)	9.50 (3.36)	6.81 (2.23)	0.076
Neutrophils (×10^3^ cells/μL)	6.31 (2.73)	3.62 (1.07)	0.026
Platelet count (×10^3^ cells/μL)	267.22 (81.54)	239.90 (63.41)	0.39
Total cholesterol concentration (mg/dL)	175.50 (44.69)	199.20 (39.04)	0.26
Triglyceride concentration (mg/dL)	110.36 (56.62)	82.45 (41.40)	0.27

**Table 2:** Demographic and clinical features of patients with acute alcohol consumption (AAC) and healthy controls. Quantitative variables are presented as mean (standard deviation), and qualitative variables as * number of cases (percentage). AST: aspartate aminotransferase; ALT: alanine aminotransferase; GGT: gamma-glutamyl transferase; ALP: alkaline phosphatase; LDH: lactate dehydrogenase. A *p* < 0.05 was considered statistically significant.

Variable	AAC Patients (*n* = 9)	Controls (*n* = 9)	*p*-Value
Age (years)	19.11 (3.36)	22.67 (2.05)	0.018
Male (n *)/Female (n *)	7 (77.78)/2 (22.22)	5 (55.56)/4 (44.44)	0.31
AST activity (U/L)	30.40 (13.17)	18.67 (4.06)	0.036
ALT activity (U/L)	18.78 (4.07)	20.78 (16.02)	0.62
ALP activity (U/L)	96.22 (51.69)	59.64 (12.50)	0.082
GGT activity (U/L)	19.79 (4.91)	15.78 (8.16)	0.26
LDH activity(U/L)	214.56 (90.16)	153.67 (17.72)	0.093
Leukocytes (×10^3^ cells/μL)	8.11 (1.66)	6.53 (3.05)	0.22

**Table 5:** Studies included in our systematic review investigating antimicrobial peptides (AMPs) in rodent models of acute (binge) alcohol exposure.

Author (Year)	Model	Sample Tissue	Antimicrobial Peptide	Analytical Technique(s)	Effect Main Findings
Wang et al. (2012) [30]	Male C57BL/6N mice (9 weeks) receiving a single ethanol dose (6 g/kg) by gavage after overnight fasting	Ileum	CRAMP	qRT-PCR	Alcohol significantly reduced CRAMP mRNA expression
Rendon et al. (2013) [28] 2/20/2026 6:08:00 PM	Male C57BL/6 mice (8–9 weeks) gavaged with alcohol (2.9 g/kg) prior to a ~12.5% total body surface area full-thickness burn	Small intestine	REG3B, REG3G	qRT-PCR	Significant post-injury reduction in expression
Lippai et al. (2014) [31]	Female C57BL/6J mice (6–8 weeks) exposed to an acute binge alcohol (Lieber-DeCarli diet, 5 g/kg for 3 consecutive days) or chronic alcohol feeding (5% ethanol for 5 weeks via oral gavage) *	Small intestine	REG3B	Western Blot, qRT-PCR	Acute alcohol binge increased mRNA and protein levels; chronic exposure decreased both
Neyrinck et al. (2016) [32]	Male C57BL/6J mice (12 weeks) fed an acute ethanol solution (30% *w*/*v*, 6 g/kg) by intragastric gavage	Colon	Lyz1, PlA2g2, DEFA, REG3	qRT-PCR	No significant changes in any AMPs after acute alcohol challenge
Hammer et al. (2017) [33]	Male C57BL/6 mice (12 weeks) gavaged with alcohol (2.9 g/kg) before a ~12.5% total body surface area full-thickness burn	Small intestine	REG3B, REG3G, LCN2	qRT-PCR	No significant differences in AMP expression with ethanol alone LCN2 transcripts were increased following the combined injury
Morishima et al. (2024) [34]	Male C57BL/6N mice (7 weeks) receiving three oral ethanol doses (5 g/kg/dose) at 12 h intervals	Colon	ANG4, ANG5, ANG6	3′ mRNA-seq transcriptomes, RT-qPCR (only for Ang4, Ang5)	No significant differences in expression

* This study was not included in Table 4 to avoid presenting overlapping results. ANG: angiogenin; CRAMP, cathelin-related antimicrobial peptide (murine LL-37 homolog); DEFA, α-defensins; DEFA-RS1: alpha-defensin-related sequence 1 precursor; ELISA: enzyme-linked immunosorbent assay; LCN2, lipocalin-2; Lyz1, Lyz2, lysozyme C-1, C-2; PlA2g2: phospholipase A2 group II; qRT-PCR, quantitative real-time polymerase chain reaction; REG3B, REG3G: regenerating islet–derived proteins 3β and 3γ.

**Table 6:** Studies included in our systematic review analyzing antimicrobial peptides (AMPs) in rodent models of chronic alcohol exposure or binge-on-chronic alcohol exposure.

Author (Year)	Model	Sample Tissue	Antimicrobial Peptide	Analytical Technique(s)	Main Findings
Yan et al. (2011) [25]	Male C57/B6 mice (8 weeks) receiving continuous intragastric ethanol infusion at increasing doses (final alcohol delivered, 30.9 g/kg/day) for 1 day, 1 week and 3 weeks.	Proximal, mid, and distal small intestine and colon	REG3B, REG3G, DEF5	qRT-PCR, Western blot, immunohistochemistry	Alcohol feeding for 1 or 3 weeks down-regulated gene and protein expression of REG3G in the proximal small intestine. DEFA5 expression levels did not differ significantly in mice after 3 weeks of enteral feeding Protein expression of REG3B and REG3G was down-regulated in the jejunum
Hartmann et al. (2013) [49]	Male C57BL/6N mice receiving continuous intragastric feeding of alcohol (Lieber DeCarli diet up to 30.9 g/kg/day) for 2 weeks	Jejunum	REG3B, REG3G, CRAMP, DEFB1	qRT-PCR, Western blot	Inhibition of REG3B and REG3G protein expression in the jejunum; CRAMP or DEFB1 mRNA expression unaffected
Shao et al. (2018) [50]	Male mice C57/BL (Cre mice, 8–10 weeks) fed Lieber-DeCarli diet, gradually exposed to alcohol (5% *w*/*v*) for 24 days	Ileum	DEFB1, DEFB2, CRAMP	qRT-PCR	Increased expression of DEFB1 and DEFB2; CRAMP reduced
Shukla et al. (2018) [51]	Female C57BL/6 mice (12–14 weeks) fed Lieber-DeCarli liquid diet with 4% ethanol for 4 weeks during the intervals and recovery period of DSS-induced colitis	Ileum, colon	DEFA4, DEFA5, DEFA6	qRT-PCR, confocal immunofluorescence microscopy	Alcohol reduced mRNA of DEFA4, DEFA5, and DEFA6 and decreased DEFA6 protein Alcohol feeding did not induce α-defensin expression in colon Chronic alcohol feeding abolished colitis-induced expression of α-defensins in colon. DSS-induced colitis also elevated expression of DEFA4 and DEFA6 genes in the ileum. Alcohol feeding reduced the expression of DEFA4, DEFA5 and DEFA6 genes in the ileum of DSS-treated mice. Confocal immunofluorescence microscopy revealed a predominant localization of DEFA6 in the crypt epithelial cells in the colon of DSS-treated mice, but it was very low in the colon of ethanol-fed DSS-colitis mice
Hendrikx et al. (2019) [53]	Female and male C57BL/6 mice (8–12 weeks) fed with Lieber–DeCarli diet for 15 days, starting at day 6 with ethanol feeding (36%) until the end. At day 16, mice were gavaged with one dose of ethanol (5 g/kg BW) in the morning and sacrificed 9 h later.	Jejunum	REG3B, REG3G, DEFA3, DEFA5, S100a8, LCN2	qRT-PCR	Reduced expression of REG3G and REG3B. Expression of DEFA3, DEFA5, S100a8 and LCN2 remained unchanged
Sadeghzadeh et al. (2019) [52]	Male Wistar rats receiving ethanol (4.5 g/kg BW) mixed in tap water (20% *w*/*v*) intragastrically once a day for 6 weeks	Epididymal	DEFB15, DEFB21, DEFB27, DEFB30	qRT-PCR	Increased expression of DEFB15 and 21; reduction in the gene expression of isoforms 27 and 30
Li et al. (2020) [21]	C57BL/6J mice (8–10 weeks) fed Lieber-DeCarli Diet (5% alcohol *w*/*v* following a bolus of ethanol (5 g/kg body weight) on day 24 by gavage before harvesting (24D+1B binge-on-chronic alcohol-feeding model).	Liver, colon, spleen, lung and epididymal white adipose tissue samples	CRAMP	Immunoblotting, ELISA, qRT-PCR	Increased CRAMP mRNA expression in the liver and spleen, decreased it in lung tissue; no change in the epididymal white adipose tissue. Spleen CRAMP protein was decreased, while liver levels of CRAMP were increased Serum CRAMP levels unchanged
Zhong et al. (2020) [36]	Male C57BL/6J mice (12 weeks) fed ethanol-containing Lieber-DeCarli liquid diet (up to 4.42 *w*/*v*) for 8 weeks	Ileum	DEFA1, DEFA2, DEFA4, DEFA5, DEFA20, DEFA21, DEFA24	PCR	Reduced mRNA levels of DEFA2, DEFA4, DEFA5, DEFA20, DEFA21, and reduced protein levels of DEFA5
Gu et al. (2021) [50]	C57BL/J mice (6 to 10 weeks) fed Lieber-DeCarli diet containing 5% alcohol (vol/vol) plus a bolus of ethanol (5 g/kg body weight) by gavage on day 10, 9 h before harvesting	Ileum	REG3B, REG3G	qRT-PCR	mRNA levels of REG3B or REG3G were slightly or significantly decreased by alcohol, respectively
^†^ McMahan et al. (2021) [37]	Young (4–5 months) and aged (21–22 months) BALB/cBy female mice exposed to 3 sequential daily ethanol gavages (1.5 g/kg and 1.25 g/kg, respectively) on 3 consecutive days a week for 4 weeks (3 days on ethanol, 4 days off ethanol)	Ileum	REG3B, REG3G	qRT-PCR	Increased expression of REG3B and REG3G in young mice but not in aged mice
Wang et al. (2021) [35]	C57BL/6 WT mice with induction of AH by following a chronic-binge ethanol-feeding protocol (Lieber-DeCarli diet containing 5 to 6% ethanol vol/vol for 10 or 14 days) plus a single dose of ethanol (5 g/kg of bodyweight) on day 11 or 15	Small intestines	REG3B, REG3G, DEFA	qRT-PCR	Suppression of all AMPs
Xia et al. (2021) [39]	ICR male mice (6 weeks) fed with alcohol-containing Lieber-DeCarli high-fat liquid diet at an increasing dose (2 to 6 g/kg) for 30 days	Colon	REG3B, REG3G	Western blot	Both proteins were downregulated
^‡^Yue et al. (2021) [40]	Male WT C57BL/6J mice (12 weeks) fed alcohol Lieber-DeCarli liquid diet (up to 4.42 *w*/*v*) for 8 weeks	Ileum	REG3B, REG3G, DEFA2, DEFA4, DEFA5, DEFA20	qRT-PCR	Downregulation of all AMPs
Das et al. (2022) [41]	C57BL/6J mice fed ethanol Lieber-DeCarli diet for 6 weeks	Jejunum	REG3B, REG3G	qRT-PCR, Western blot (REG3B)	No significant changes in mRNA or protein levels
Ray et al. (2022) [43]	C57BL/J mice (8 to 10 weeks) fed an alcohol-containing Lieber-DeCarli liquid diet (up to 30% of ethanol-derived calories) for 10 days	Jejunum and ileum	REG3B, REG3G	qRT-PCR (Jejunum and ileum), Western blot (Jejunum)	Reduced mRNA and protein levels
^‡^Hao et al. (2023) [44]	Male WT C57BL/6J mice (12 weeks) fed alcohol Lieber-DeCarli liquid diet for 8 weeks (increasing from 4.5% by 0.37% every 2 weeks, reaching 5.6%for the last 2 weeks)	Ileum	DEFA5, REG3B, REG3G	qRT-PCR	Downregulation of all AMPs
^†^ McMahan et al. (2023) [42]	Young (4–5 months) and aged (21–22 months) BALB/cBy female mice exposed to 3 sequential daily ethanol gavages (1.5 g/kg and 1.25 g/kg, respectively).	Ileum	DEFARS1, REG3B, REG3G, Lyz1, Lyz2, DEFA1, DEFA2	qRT-PCR	DEFARS1 and REG3G were downregulated in aged mice and upregulated in young rodents. Transcriptional levels of Lyz1/2 and DEFA1/2 remained unchanged
^‡^Yue et al. (2023) [45]	Male WT C57BL/6J mice (12 weeks) fed alcohol Lieber-DeCarli liquid diet (up to 4.42 *w*/*v*) for 8 weeks	Ileum	REG3B, REG3G, DEFA4, DEFA5, DEFA24	qRT-PCR, immunofluorescence staining of DEFA5	Reduced mRNA expression of REG3B, REG3G and a-defensins. Decreased protein DEFA5 in the small intestinal crypts
Suresh et al. (2024) [46]	C57BL/J mice (12 weeks) fed an alcohol-containing Lieber-DeCarli liquid diet for 8 weeks	Small intestine	DEFA20, DEFA23, DEFA29, DMBT1	Laser Capture Microdissection of crypts and villus tissues; proteomics	Downregulation of all AMPs.
Gao et al. (2025) [48]	Male C57BL/6J mice (8 weeks) fed Lieber-DeCarli alcoholic liquid diet (5%, *v*/*v*) for 10 days, with daily gavage of 12.5% glycerol/0.9% NaCl and 31.5% alcohol on day 20	Colon	REG3B, REG3G	qRT-PCR	Downregulation of REG3B and REG3G
Zhao et al. (2025) [47]	C57BL/6 mice (10–12 weeks) fed the Lieber-DeCarli ethanol liquid diet model for 14 days and one dose of ethanol (5g/kg body weight) in the morning of day 15	Ileum	REG3B, REG3G	qRT-PCR	Chronic-binge alcohol intake resulted in lower levels of REG3B and REG3G

^†^ McMahan et al. (2021) [37] and McMahan et al. (2023) [42] reported overlapping findings derived from the same experiment; the 2023 report included an expanded AMP panel. ^‡^Yue et al. (2021) [40], Hao et al. (2023) [44], and Yue et al. (2023) [45] presented overlapping results from a shared experimental dataset. CRAMP: cathelin-related antimicrobial peptide (murine LL-37 homolog); DEFA: α-defensins; DEFA-RS1: alpha-defensin-related sequence 1 precursor; DMBT1: deleted in malignant brain tumors 1; ELISA: enzyme-linked immunosorbent assay; LCN2: lipocalin-2; Lyz1, Lyz2: lysozyme C-1, C-2; qRT-PCR: quantitative real-time polymerase chain reaction; REG3B, REG3G: Regenerating islet–derived proteins 3β and 3γ.

## Data Availability

The data presented in this study are available from the corresponding author upon reasonable request and are not publicly available due to ethical restrictions.

## Supplementary Materials

The following supporting information can be downloaded at: https://www.mdpi.com/article/10.3390/ijms27042026/s1.

## Abbreviations

The following abbreviations are used in this manuscript:

18S rRNA	18S ribosomal RNA
AAC	Acute alcohol consumption
AMPs	Antimicrobial peptides
AUD	Alcohol use disorder
BPI	Bactericidal/permeability-increasing protein
DEFA1-3	Alpha-defensin 1-3
DEFB1-3	Beta-defensin 1-3
LBP	Lipopolysaccharide-binding protein
LCN2	Lipocalin-2
LEAP-1/2	Liver-expressed antimicrobial peptide 1/2
NIAAA	National Institute on Alcohol Abuse and Alcoholism
PB	Peripheral blood
qPCR	Quantitative polymerase chain reaction
REG3A/G	Regenerating family member 3 alpha/gamma
