# Supplementary material for: Analysis of Antimicrobial Peptide Expression Under Acute and Chronic Alcohol Exposure: A Cross-Sectional Study and a Systematic Review of the Literature
Source: Int J Mol Sci. 2026 Feb 20;27(4):2026. doi: 10.3390/ijms27042026 (PMC12940937; doi:10.3390/ijms27042026)
Supplement: Supplementary file 1 [file ijms-27-02026-s001.zip › ijms-4091031-supplementary.pdf]

Analysis of antimicrobial peptide expression under acute and chronic alcohol exposure: a cross-sectional study and a systematic review of the literature

Supplementary Material

Maura Rojas-Pirela [1,2,3#], Cristian Herrera Flores [1,2,4#], Pilar Costa-Alba[5], María-Lourdes Aguilar [2,6], David Puertas-Miranda [1,3], Beatriz Cicuéndez[7], María-Ángeles Pérez-Nieto [1,2] Cintia Folgueira[7], Daniel Salete-Granado[1,2,3], Alfonso Mora[7], Guadalupe Sabio[7], and Miguel Marcos [1,2,3*]

[1]Department of Internal Medicine, University Hospital of Salamanca, Salamanca, Spain

[2]Institute of Biomedical Research of Salamanca (IBSAL), Salamanca, Spain

[3]Department of Medicine. University of Salamanca (USAL), Salamanca, Spain

[4]Department of Cardiology. University Hospital of Salamanca (USAL). Centro de Investigación Biomédica en Red de Enfermedades Cardiovasculares (CIBER-CV). Salamanca, Spain

[5]Primary Care Management of Salamanca, Castilla and León Health Service (SACyL), Salamanca, Spain.

[6]Department of Psychiatry, University Hospital of Salamanca, Salamanca, Spain

[7]Organ Crosstalk in Metabolic Diseases Group, Molecular Oncology Program, Spanish National Cancer Centre (CNIO), Madrid, Spain

# These authors contributed equally as first authors of this article.

*Correspondence: mmarcos@usal.es (M.M.) and mrojaspirela@usal.es (M.R.-P.).

Keywords: Antimicrobial peptides, alcohol, immune response, altered expression

Supplementary Table 1. Forward and reverse primer sequences used for qRT-PCR.

Gene	Forward	Reerse
**Human**		
Cathelicidin (LL-37)	5´-GCTCCTTTGACATCAGTTG-3´	5´-GTCCTGGGTACAAGATTCC-3´
Bactericidal permeability-increasing protein (BPI)	5´-CAGGTCTGCGAGAAAGTGAC-3´	5´-AAAGGGAGGTGGATTGTGG-3´
Lipopolysaccharide binding protein (LBP)	5´-CTGCTGGCATTGCTGCTTAC-3´	5´-AGGCTGTGGAACTCATAGCG-3´
18S ribosomal RNA (18S rRNA)	5´-GGATGAGGTGGAACGTGTGA-3´	5´-CCAGACCATTGGCTAGGACC-3´
**Mouse**		
CRAMP/LL-37_ mouse	5´-GGCGGTCACTATCACTGCTG-3´	5´-TCGGAACCTCACAGACTTGG-3´
Liver-expressed antimicrobial peptide 1 (LEAP-1)	5´-CCTGAGCAGCACCACCTATC-3´	5´-GCAACAGATACCACACTGGGA-3´
Liver-expressed antimicrobial peptide 2 (LEAP-2)	5´-CTGCTGGGTCAGGTCAATAGT-3´	5´-GTATGGACATCACTCCTGGGC-3´
Regenerating family member 3 alpha (REG3A)	5´-GCTTATCGCTCCCACTGCTA-3´	5´-GGTAGTTGTCCACTCTGCCG-3´
Regenerating islet-derived protein 3-gamma (REG3G)	5´-CCGACACTGGGCTATGAACC-3´	5´-TGGGATCTTGCTTGTGGCTA-3´
Alpha-defensin 1 (DEFA1)	5´-GCTGCCTGCTCATCCTAATCCAT-3	5´-GGATAGAATCAGCCTGGACCTG-3´
Alpha-defensin 2 (DEFA2)	5´-GCTCAGGACGACTTTTGTTC	5´-CAATTTATTGAGAAGTGGTTATCA-3´
Alpha-defensin 3 (DEFA3)	5´-TCGCTGAACATGGAGACCAC	5´-CGAGGTAGTCATCAGGCACC
B-defensin 2 (DEFB2)	5´-TGGAGTCTGAGTGCCCTTTC-3´	5´-AGTGGTCAAGTTCTGCTTCGT-3´
B-defensin 1 (DEFB1)	5´-ACATCTGCCTGGTCCTGAGT-3´	5´-GGAAGCCTGTGTACCGTGTT-3´
LPB_mouse	5´GACCTGGACTTGACTCCGTC-3´	5´ACAGTGCCCGCTCTTAAAGT-3´
Lipocalin-2 (LCN2)	5´GCCCTGAGTGTCATGTGTCT-3´	5´-ACAACGTACCACCTGCCCC-3´
TATA-box binding protein (TBP)	5´-GAAGCTGCGGTACAATTCCAG-3´	5´-CCCCTTGTACCCTTCACCAAT-3´
Hypoxanthine phosphoribosyltransferase (Hprt)	5´-GCCTTCCCGCTCTACTGAAA-3´	5´-GCTCAGCACACCTACCTACG-3´
Serine and arginine-rich splicing factor 4 (SRSF4)	5´-GCCTTCCCGCTCTACTGAAA-3´	5´-GCTCAGCACACCTACCTACG-3´

Supplementary Section S1. Search strategies

Scopus on 22-11-2025:

#1	("antimicrobial peptide" OR "AMPs" OR "host defense peptide" OR "HDPs" OR "bactericidal peptide" OR "antimicrobial cationic peptide" OR "defensin" OR "cathelicidin" OR “REG3” OR “regenerating islet-derived protein 3”)	
#2	("alcohol" OR "ethanol" OR "alcohol use disorder" OR “Alcohol-Related Disorders" OR "alcohol exposure" OR "alcohol abuse" OR "chronic alcoholic intoxication" OR "alcohol dependence" OR "alcohol addiction" OR “alcohol intake” OR "acute alcohol consumption" OR "excessive ethanol intake" OR “alcoholism" OR "binge drinking" OR "alcoholic intoxication" OR "Alcohol Drinking" OR “Alcohol Misuse”)	
#3	("biomarker" OR “expression”)	
#4	#1 AND #2 AND #3	1680

Web of Science on 22-11-2025:

#1	("antimicrobial peptide" OR "AMPs" OR "host defense peptide" OR "HDPs" OR "bactericidal peptide" OR "antimicrobial cationic peptide" OR "defensin" OR "cathelicidin")	
#2	("alcohol" OR "ethanol" OR "alcohol use disorder" OR “Alcohol-Related Disorders" OR "alcohol exposure" OR "alcohol abuse" OR "chronic alcoholic intoxication" OR "alcohol dependence" OR "alcohol addiction" OR “alcohol intake” OR "acute alcohol consumption" OR "excessive ethanol intake" OR “alcoholism" OR "binge drinking" OR "alcoholic intoxication" OR "Alcohol Drinking" OR “Alcohol Misuse”)	
#3	("biomarker" OR “expression”)	
#4	#1 AND #2 AND #3	89

Pubmed on 22-11-2025:

#1	"antimicrobial peptide*"[tiab] OR "AMPs"[tiab] OR "host defense peptide*"[tiab] OR "HDPs"[tiab] OR "bactericidal peptide*"[tiab] OR "antimicrobial cationic peptide*"[tiab] OR "defensin*"[tiab] OR "cathelicidin*"[tiab] OR "gene expression"[tiab] OR "expression profile*"[tiab] OR "expression analys*"[tiab] OR "mRNA expression"[tiab] OR "Antimicrobial Cationic Peptides"[Mesh] OR "Defensins"[Mesh] OR "Cathelicidins"[Mesh] OR "Gene Expression"[Mesh]
#2	("alcohol*"[tiab] OR "ethanol"[tiab] OR "alcohol use disorder"[tiab] OR "AUD"[tiab] OR "alcohol consumption"[tiab] OR "alcohol intake"[tiab] OR "alcohol exposure"[tiab] OR "alcohol abuse"[tiab] OR "alcohol dependence"[tiab] OR "alcohol

	addiction"[tiab] OR "alcoholism"[tiab] OR "alcoholic intoxication"[tiab] OR "chronic alcoholic intoxication"[tiab] OR "binge drinking"[tiab] OR "alcohol misuse"[tiab] OR "Alcohol Drinking"[Mesh] OR "Alcoholism"[Mesh] OR "Alcohol-Related Disorders"[Mesh] OR "Ethanol"[Mesh] OR "Alcoholic Intoxication"[Mesh] OR "Binge Drinking"[Mesh])	
#3	("biomarker*"[tiab] OR "Biomarkers"[Mesh] OR "Biological Markers"[Mesh])	
#4	#1 AND #2 AND #5	1102
